# Supplementary material for: In situ acid etching boosts mercury accommodation capacities of transition metal sulfides
Source: Nat Commun. 2023 Mar 13;14:1395. doi: 10.1038/s41467-023-37140-5 (PMC10011380; doi:10.1038/s41467-023-37140-5)
Supplement: Supplementary file 1 — Supplementary Information [file 41467_2023_37140_MOESM1_ESM.pdf]

Supplementary Information

In situ acid etching boosts mercury accommodation capacities of transition metal sulfides

Hailong Li[1], *Jiaoqin Zheng*[1], *Wei Zheng*[1], *Hongxiao Zu*[1], *Hongmei Chen*[1], *Jianping Yang*[1], *Wenqi Qu*[1], *Lijian Leng*[1], *Yong Feng*[2], *Zequn Yang*[1*]

1. School of Energy Science and Engineering, Central South University, Changsha, 410083, China

2. Environmental Research Institute, South China Normal University, Guangzhou, 510631, China

*To whom correspondence should be addressed:

TEL: +86-15675117541

E-mail: Zequn_Yang@hotmail.com

List of Supplementary Tables:

Supplementary Table 1. Hg^0 adsorption capacities of different metal sulfides

Supplementary Table 1. Hg^0 adsorption capacities of different metal sulfides

Sorbents	breakthrough threshold	Hg^0 adsorption capacities ($mg\ g^{-1}$)	References
CuS	50%	52.88	[1]
ZnS	50%	0.498	[2]
FeS_x	4%	0.22	[3]
CoS_x	25%	2.07	[4]
MoS_2	90%	16.26	[5]
PbS	NA	2.76	[6]
Mn-SnS_2	NA	0.57	[7]

List of Supplementary Figures:

Supplementary Figure 1. A diagrammatic illustration of the sample preparation procedures

Supplementary Figure 2. XRD patterns of different samples

Supplementary Figure 3. A diagrammatic illustration of the fixed-bed reaction system

Supplementary Figure 4. Mercury removal performances of ZnS in-situ etched by different concentrations of H_2SO_4

Supplementary Figure 5. Textural property comparison among different samples

Supplementary Figure 6. Isothermal N_2 absorption-desorption patterns of different samples

Supplementary Figure 7. TEM images of (a) pristine ZnS, (b) 0.368-ZnS, and (c) ex0.368-ZnS

Supplementary Figure 8. XPS patterns of Zn *2p* and S *2p* of different samples

Supplementary Figure 9. Selected areas for different samples to take the EDS spot scanning

Supplementary Figure 10. EDS patterns of different selected areas as shown in Figure S9

Supplementary Figure 11. EDS mapping patterns of different samples

Supplementary Figure 12. A diagrammatic illustration of the nested-tube reactor

Supplementary Figure 13. Hg-TPD patterns of Hg-laden pristine ZnS, 0.368-ZnS, and ex0.368-ZnS being treated in a nested-tube reactor. The error bars in the inserted figure correspond to the standard deviation of three independent measurements, and the data are presented as mean values

Supplementary Figure 14. The XPS pattern comparisons between (a) Hg-laden 0.368-ZnS and Hg-laden pristine ZnS; and (b) & (c) fresh 0.368-ZnS and Hg-laden 0.368-ZnS

Supplementary Figure 15. The crystal facet distances of ZnS along different directions

Supplementary Figure 16. Top- and side-view of the slab model of ZnS(111) surface (violet: Zn, pink: S)

Supplementary Figure 17. The slab model of ZnS(111) surface with S or Zn vacancy (violet: Zn, pink: S)

Supplementary Figure 18. Possible Hg adsorption sites on intact, S-defect, and Zn-defect surfaces (violet: Zn, pink: S; (i) hollow, (ii) Zn-top, (iii) Se-top, (iv) bridge, (v) S-defect, (vi) under-coordinated Zn-top, (vii) Zn-defect, and (viii) under-coordinated S-top sites)

Supplementary Figure 19. Partial density of state (PDOS) analysis of the interactions between Hg and (a) intact ZnS(111), (b) S-defect ZnS(111), and (c) Zn-defect(111) surfaces

Supplementary Figure 20. STEM-HAADF images of pristine and acid-etched ZnS (fresh samples)

Supplementary Figure 21. Hg^0 accommodation capacity of ex0.368-ZnS (inserted with its real-time Hg^0 removal efficiency)

Supplementary Figure 22. The Hg^0 breakthrough curves of (a-d) pristine CoS, NiS, CdS, and MoS_2, and (e-h) acid-etched CoS, NiS, CdS, and MoS_2

Supplementary Figure 23. Metal mercury recycled from Hg-laden 0.368-ZnS

Supplementary Figure 1. A diagrammatic illustration of the sample preparation procedures

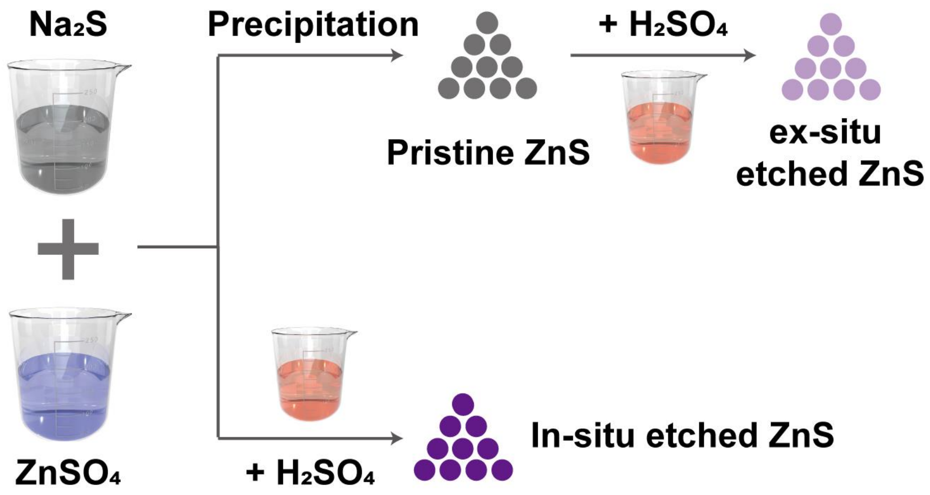

Supplementary Figure 2. XRD patterns of different samples

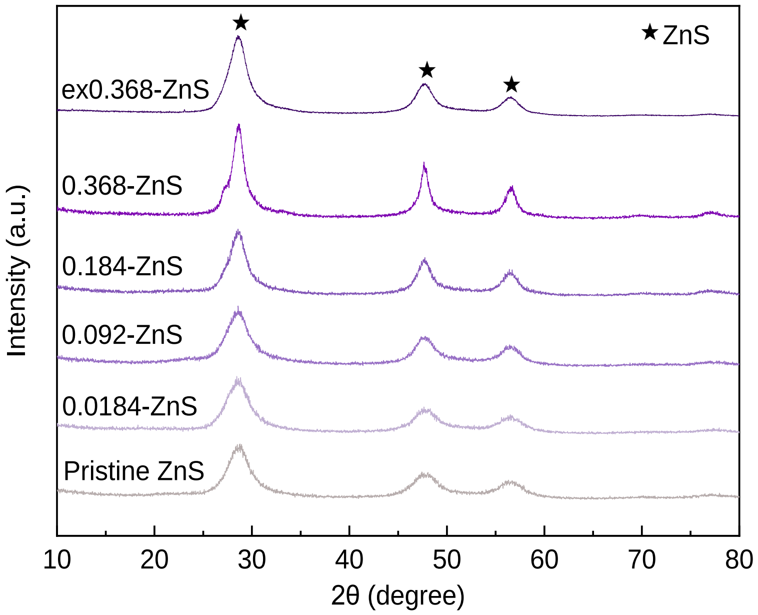

Supplementary Figure 3. A diagrammatic illustration of the fixed-bed reaction system

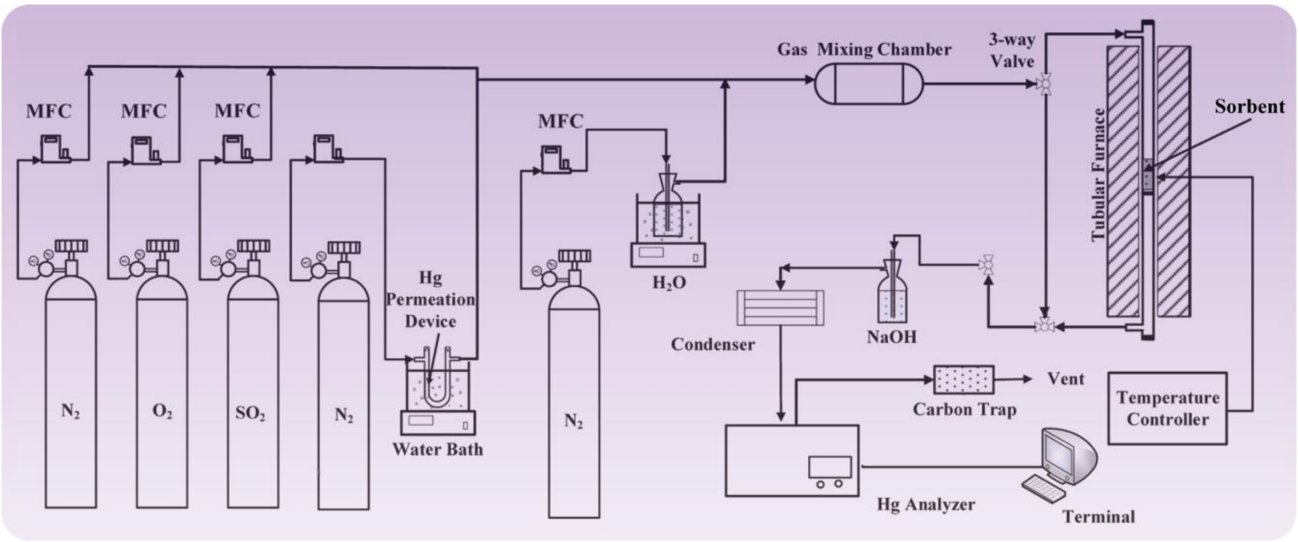

Supplementary Figure 4. Mercury removal performances of ZnS in-situ etched by different concentrations of H_2SO_4

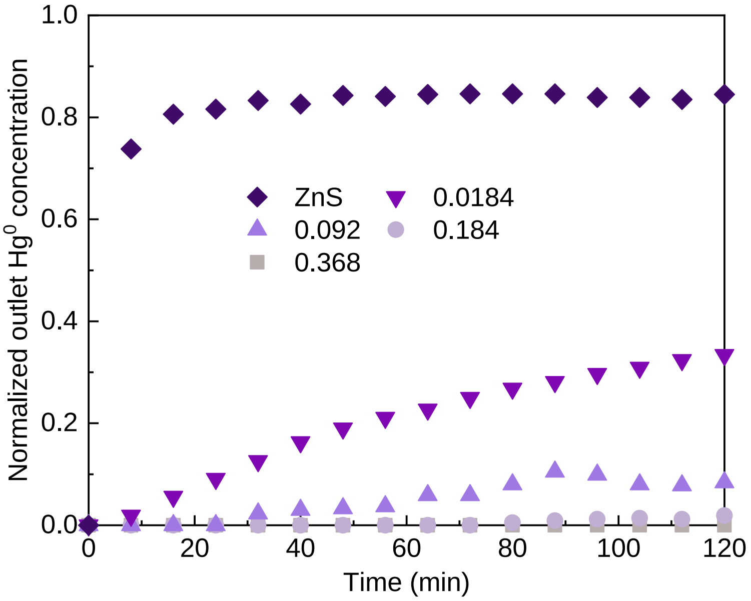

Supplementary Figure 5. Textural property comparison among different samples

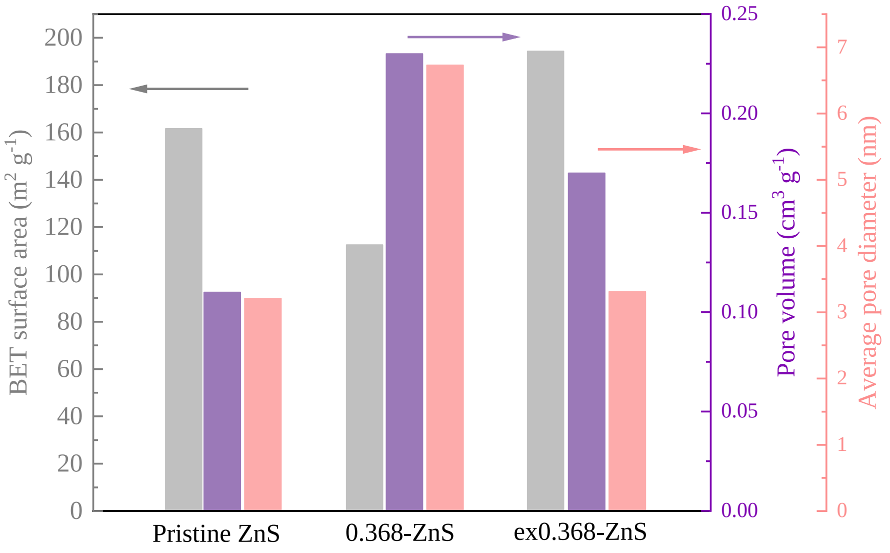

Supplementary Figure 6. Isothermal N_2 absorption-desorption patterns of different samples

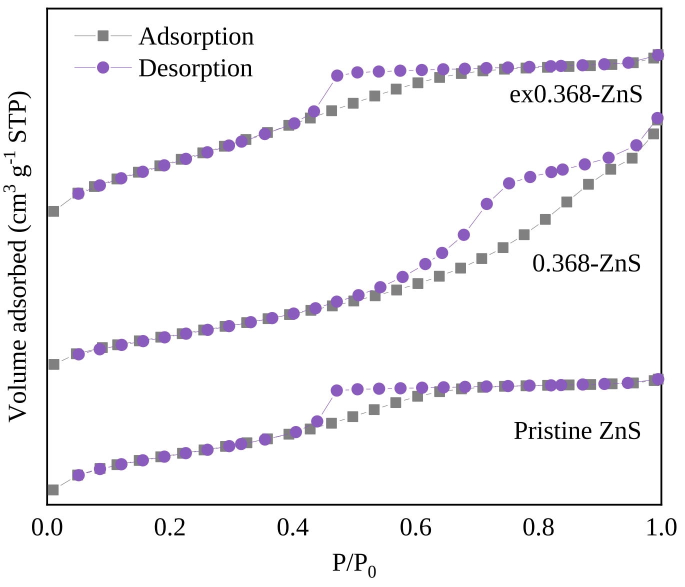

Supplementary Figure 7. TEM images of (a) pristine ZnS, (b) 0.368-ZnS, and (c) ex0.368-ZnS

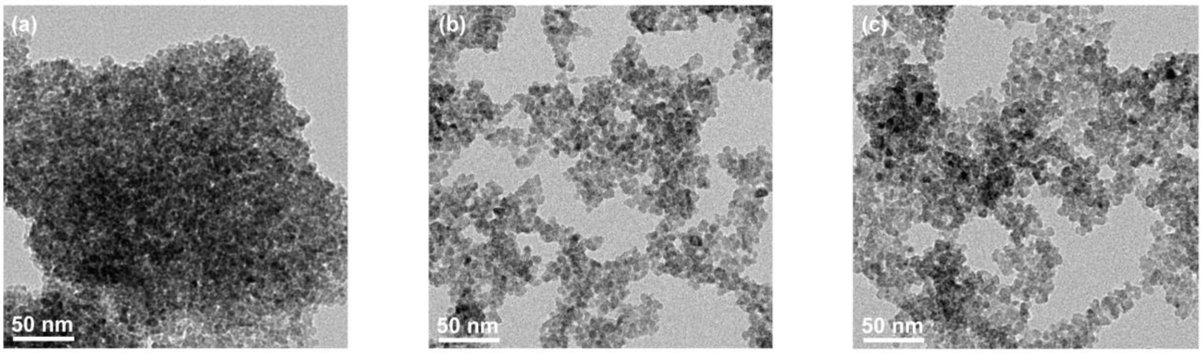

Supplementary Figure 8. XPS patterns of (a) Zn *2p* and (b) S *2p* of different samples

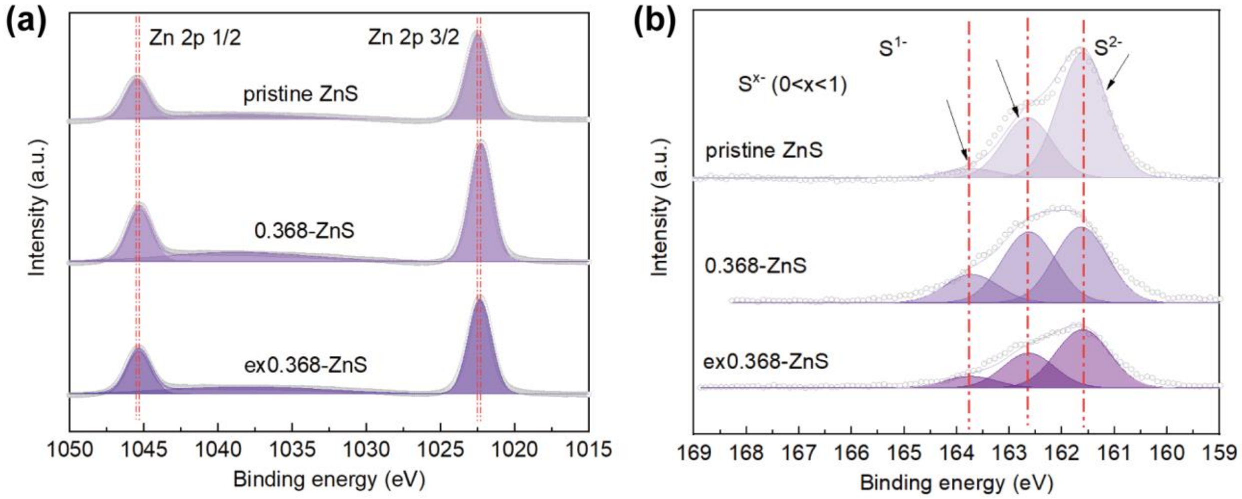

Supplementary Figure 9. Selected areas for different samples to take the EDS spot scanning

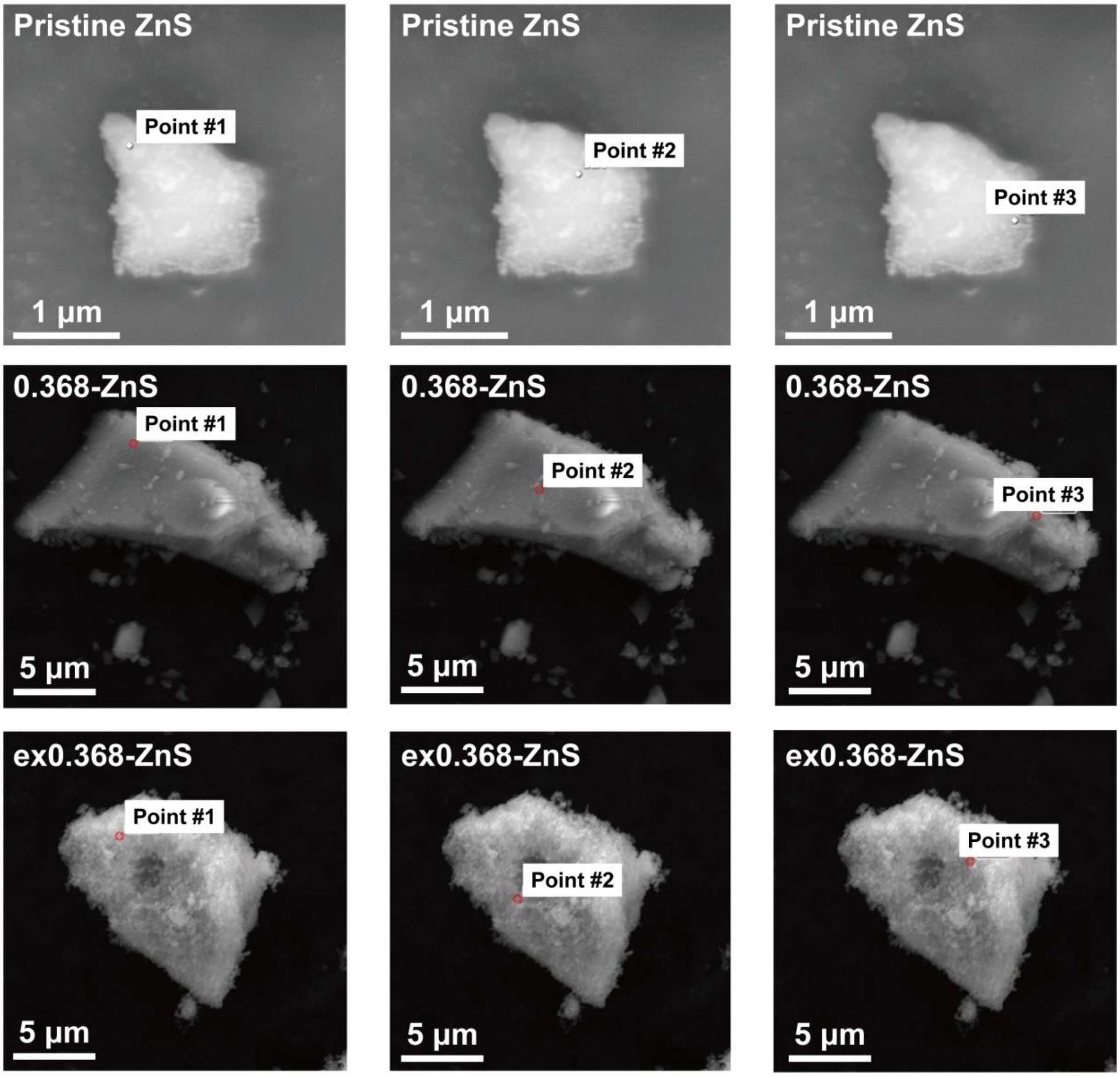

Supplementary Figure 10. EDS patterns of different selected areas as shown in Supplementary Figure 9

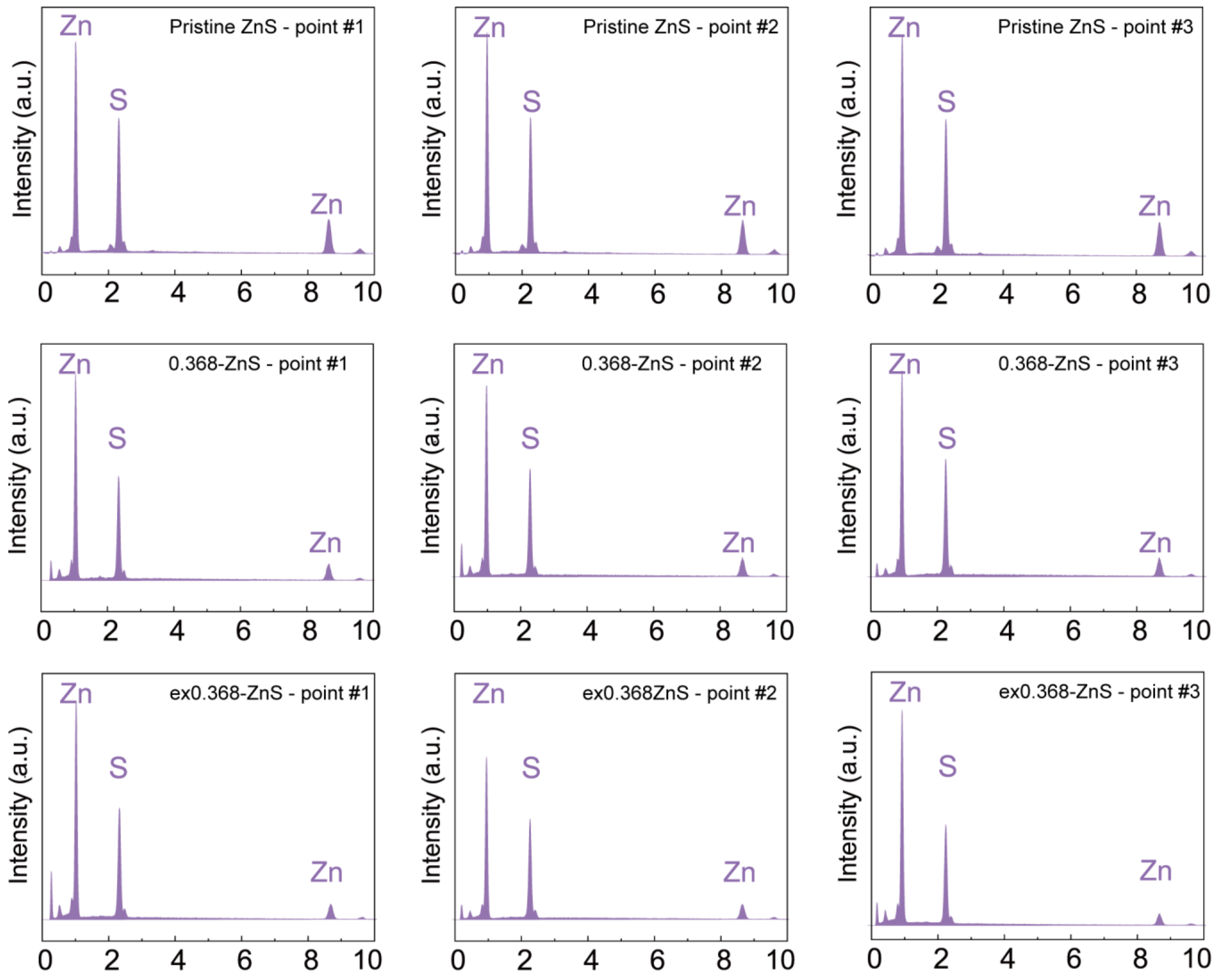

Supplementary Figure 11. EDS mapping patterns of different samples

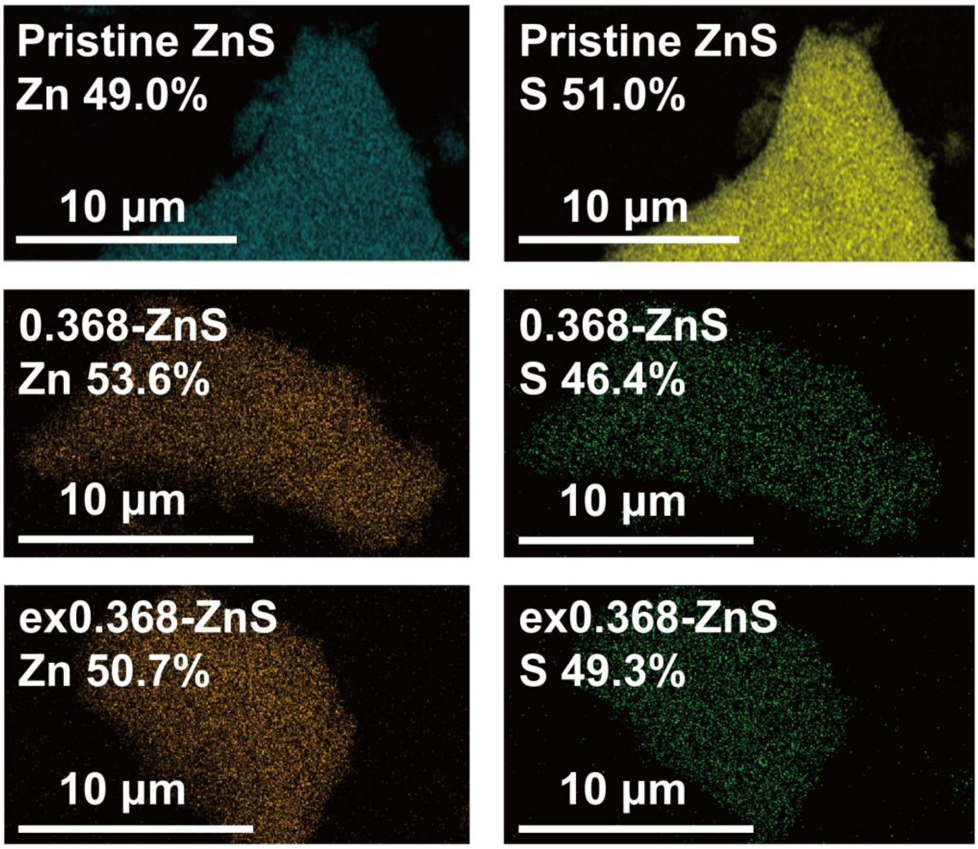

Supplementary Figure 12. A diagrammatic illustration of the nested-tube reactor

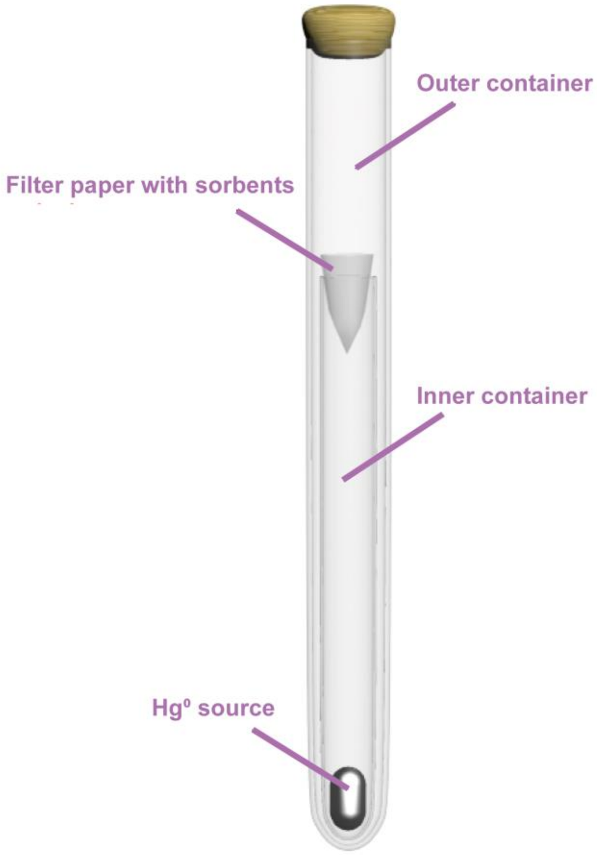

Supplementary Figure 13. Hg-TPD patterns of Hg-laden pristine ZnS, 0.368-ZnS, and ex0.368-ZnS being treated in a nested-tube reactor. The error bars in the inserted figure correspond to the standard deviation of three independent measurements, and the data are presented as mean values.

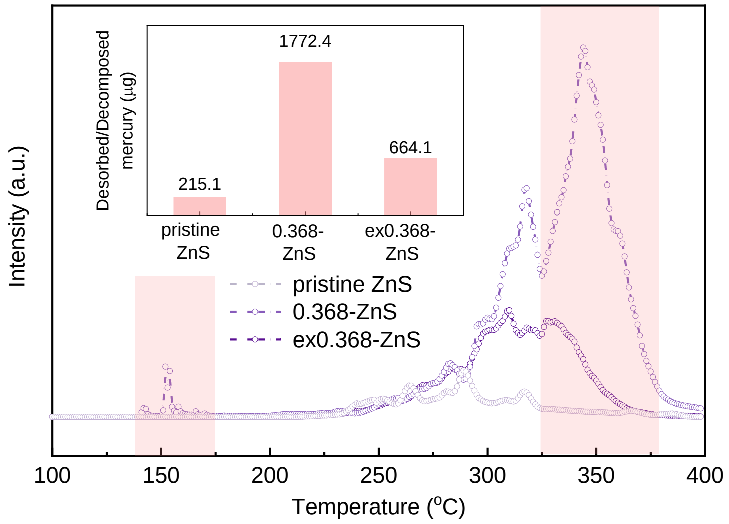

Supplementary Figure 14. The XPS pattern comparisons between (a) Hg-laden 0.368-ZnS and Hg-laden pristine ZnS; and (b) & (c) fresh 0.368-ZnS and Hg-laden 0.368-ZnS

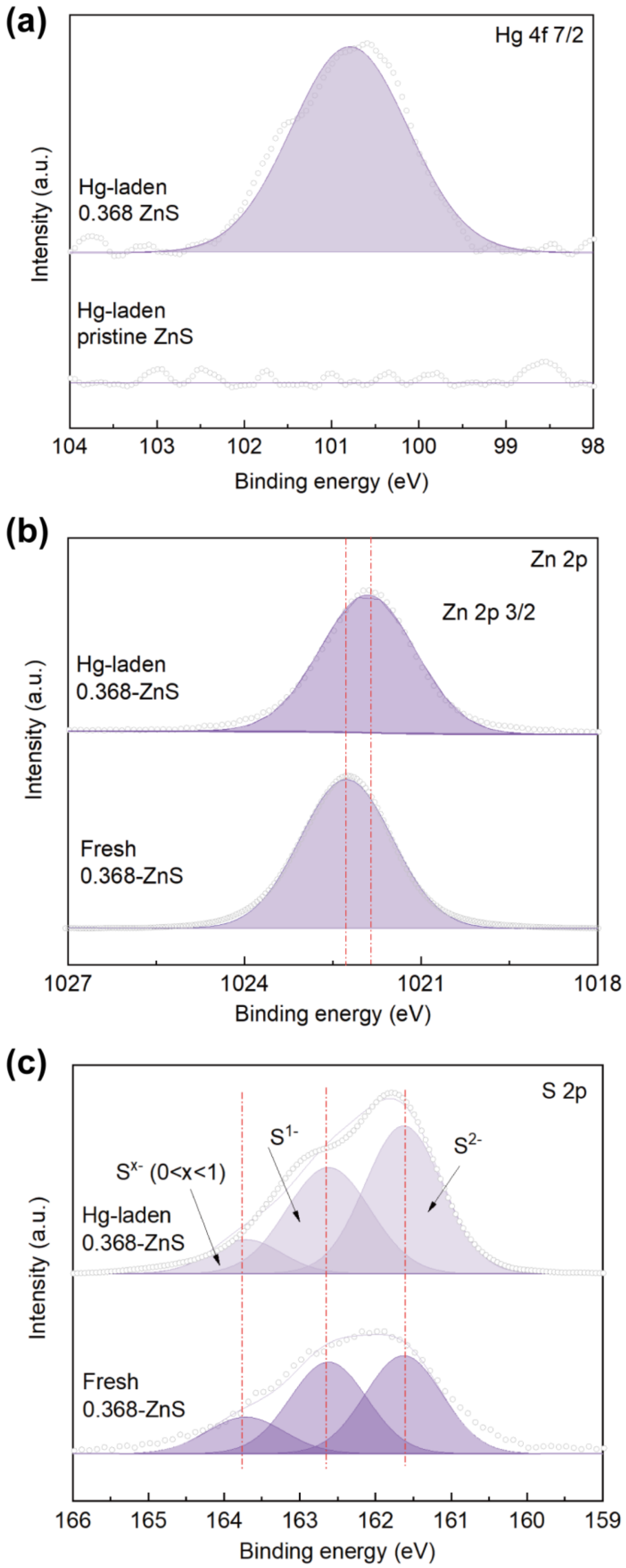

Supplementary Figure 15. The crystal facet distances of ZnS along different directions

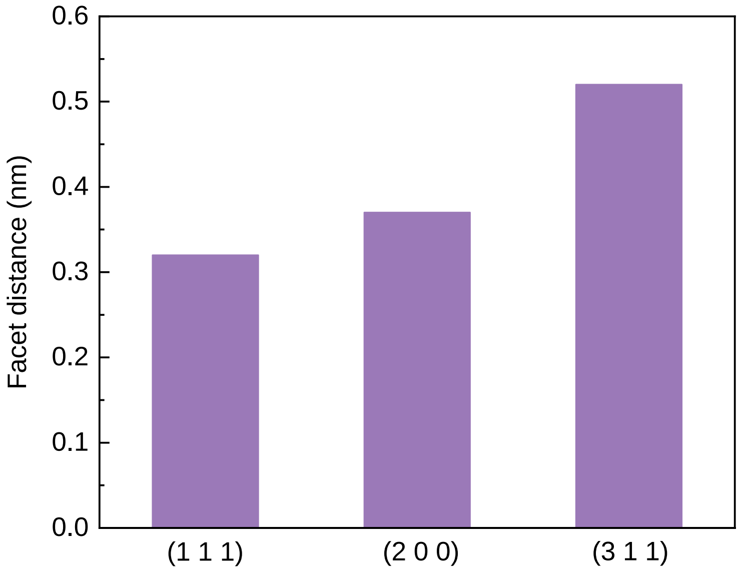

Supplementary Figure 16. Top- and side-view of the slab model of ZnS(111) surface (violet: Zn, pink: S)

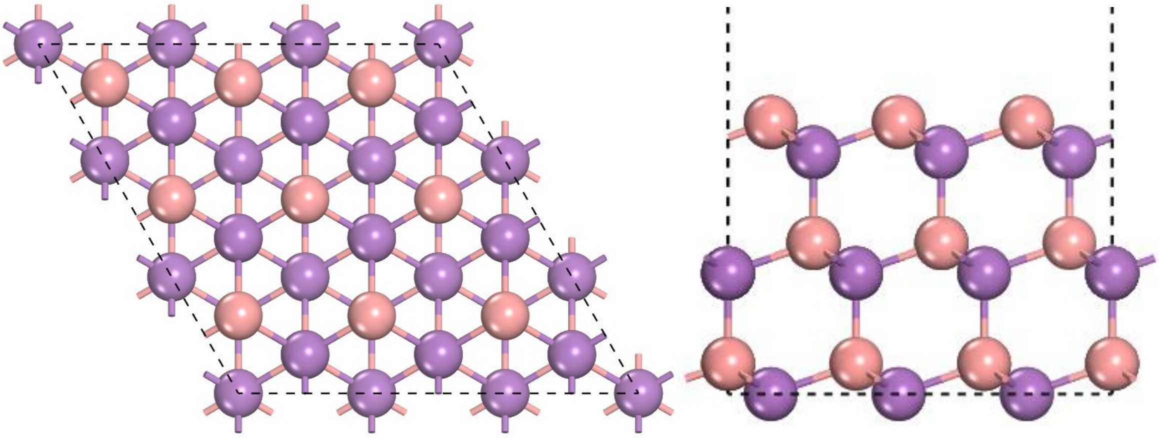

Supplementary Figure 17. The slab model of ZnS(111) surface with S or Zn vacancy (violet: Zn, pink: S)

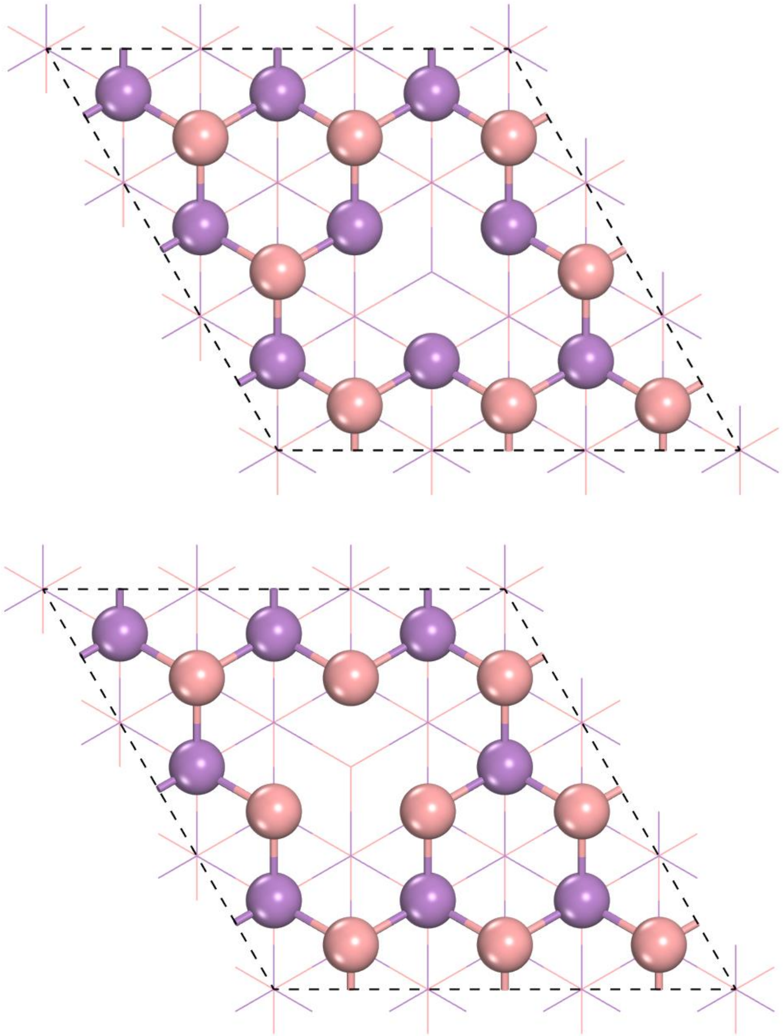

Supplementary Figure 18. Possible Hg adsorption sites on intact, S-defect, and Zn-defect surfaces (violet: Zn, pink: S; (i) hollow, (ii) Zn-top, (iii) Se-top, (iv) bridge, (v) S-defect, (vi) under-coordinated Zn-top, (vii) Zn-defect, and (viii) under-coordinated S-top sites)

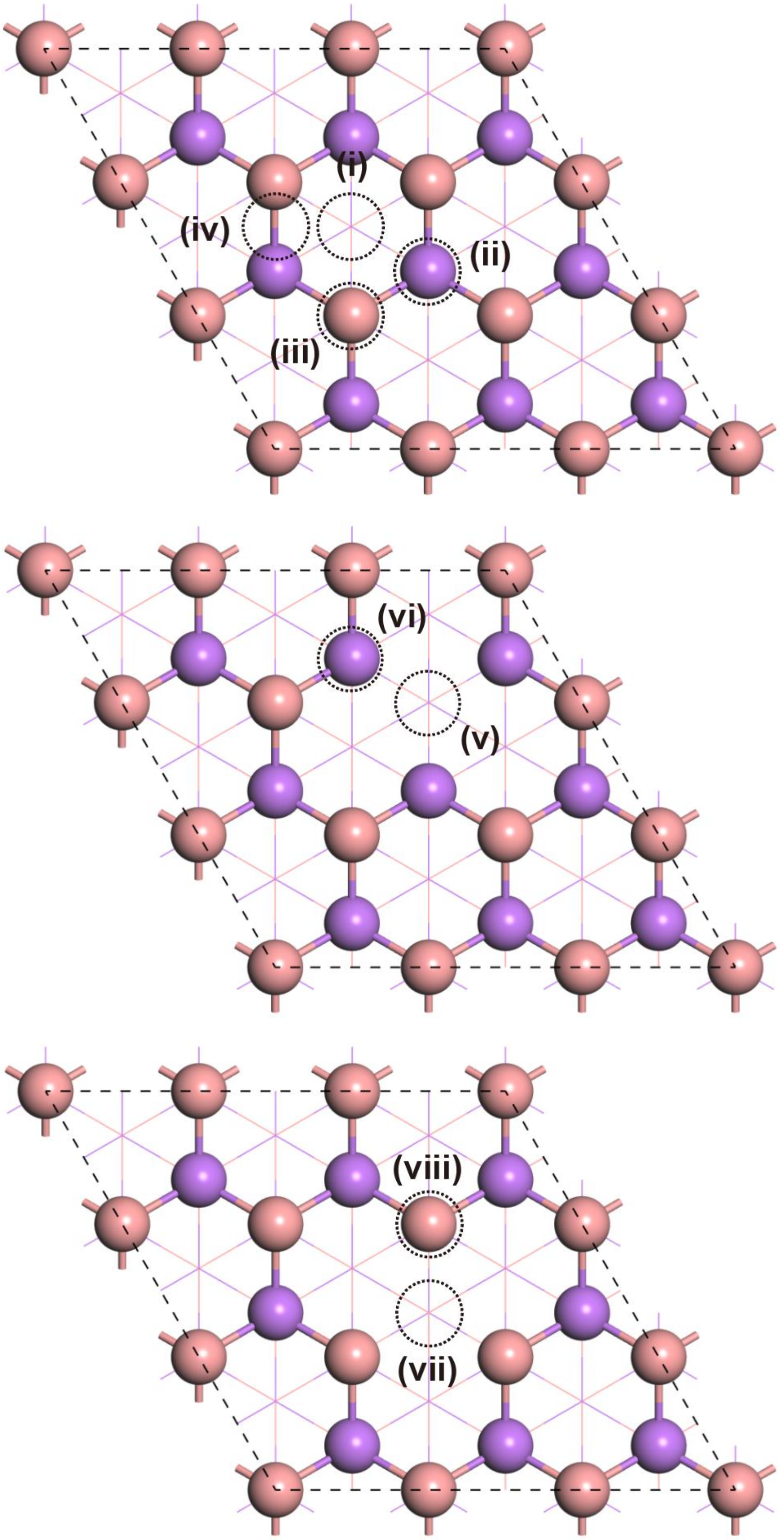

Supplementary Figure 19. Partial density of state (PDOS) analysis of the interactions between Hg and (a) intact ZnS(111), (b) S-defect ZnS(111), and (c) Zn-defect(111) surfaces

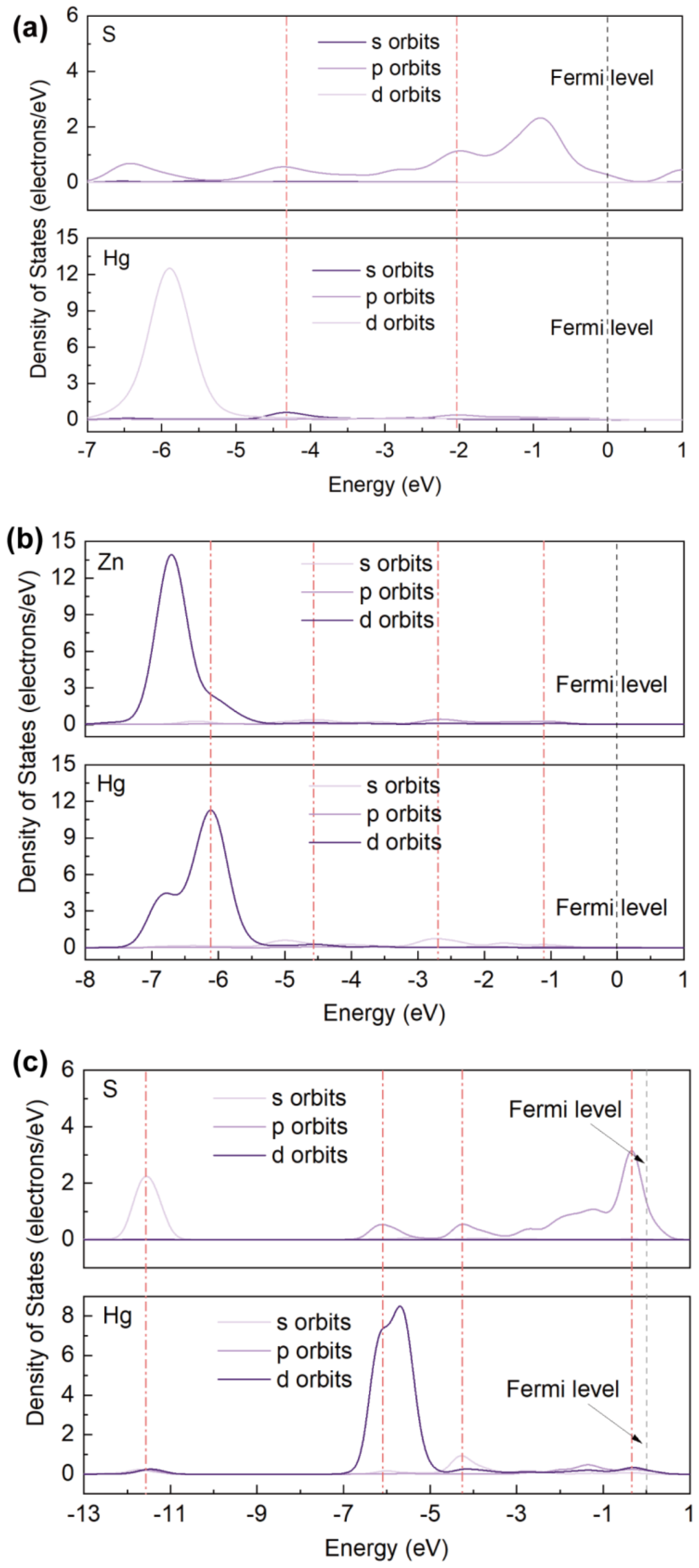

Supplementary Figure 20. STEM-HAADF images of pristine and acid-etched ZnS (fresh samples)

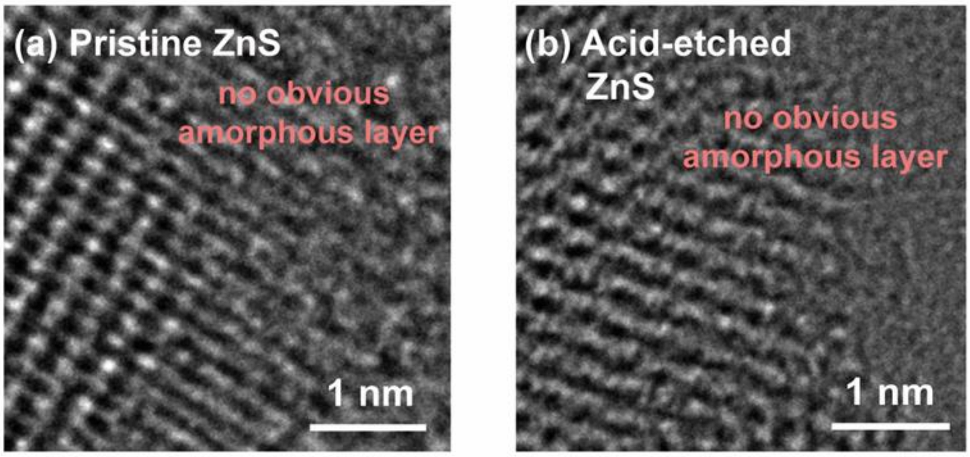

Supplementary Figure 21. Hg^0 accommodation capacity of ex0.368-ZnS (inserted with its real-time Hg^0 removal efficiency)

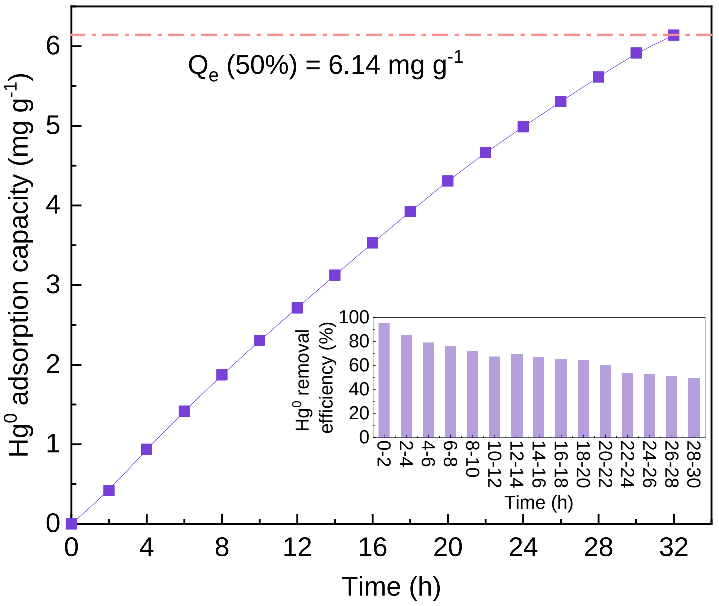

Supplementary Figure 22. The Hg^0 breakthrough curves of (a-d) pristine CoS, NiS, CdS, and MoS_2, and (e-h) acid-etched CoS, NiS, CdS, and MoS_2

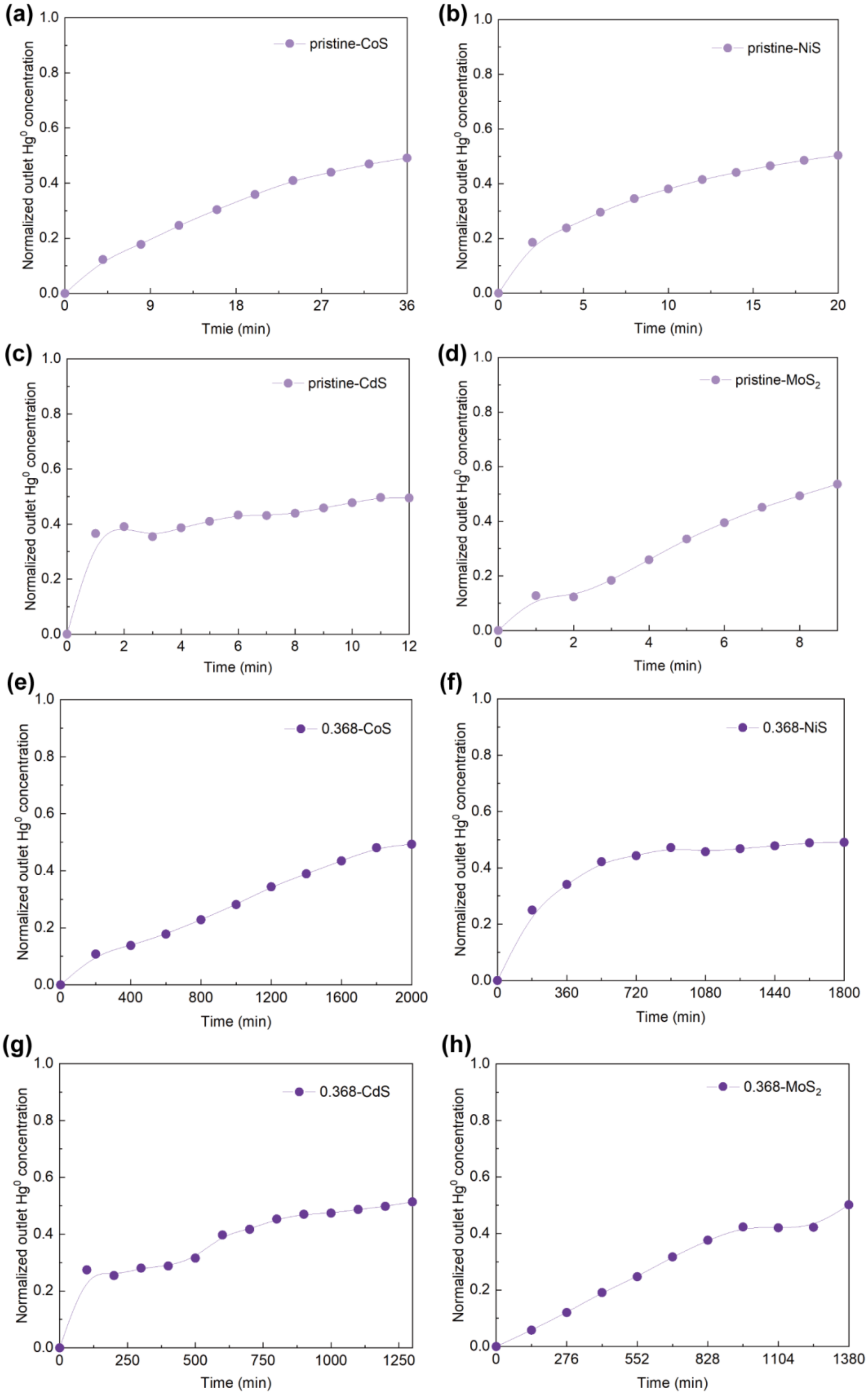

Supplementary Figure 23. Metal mercury recycled from Hg-laden 0.368-ZnS

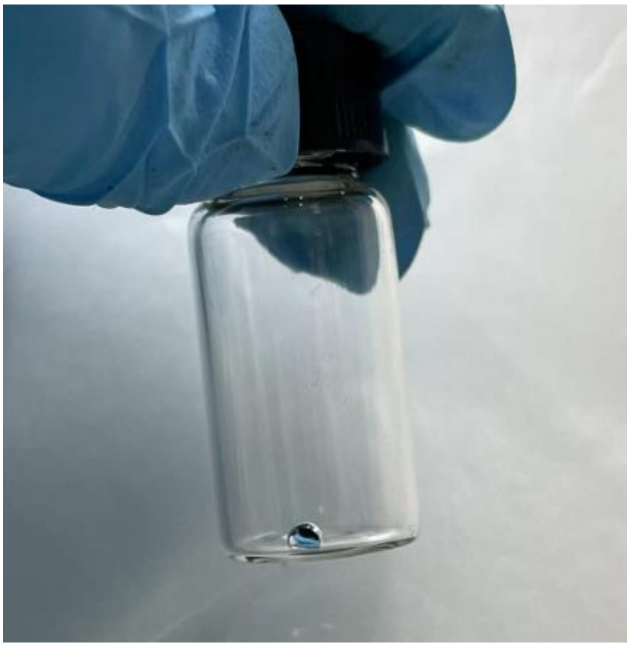

Supplementary References

1. Yang Z, *et al.* Multiform Sulfur Adsorption Centers and Copper-Terminated Active Sites of Nano-CuS for Efficient Elemental Mercury Capture from Coal Combustion Flue Gas. *Langmuir.* **34**, 8739-8749 (2018).
2. Li H, Zhu L, Wang J, Li L, Shih K. Development of Nano-Sulfide Sorbent for Efficient Removal of Elemental Mercury from Coal Combustion Fuel Gas. *Environ Sci Technol.* **50**, 9551-9557 (2016).
3. Liao Y, *et al.* Recyclable Naturally Derived Magnetic Pyrrhotite for Elemental Mercury Recovery from Flue Gas. *Environmental Science Technology.* **50**, 10562-10569 (2016).
4. Liu H, *et al.* High-efficient adsorption and removal of elemental mercury from smelting flue gas by cobalt sulfide. *Environ Sci Pollut Res Int.* **26**, 6735-6744 (2019).
5. Liu H, *et al.* Disordered MoS_2 Nanosheets with Widened Interlayer Spacing for Elemental Mercury Adsorption from Nonferrous Smelting Flue Gas. *ACS ES&T Engineering.* **1**, 1258-1266 (2021).
6. Hong Q, Liao Y, Xu H, Huang W, Qu Z, Yan N. Stepwise Ions Incorporation Method for Continuously Activating PbS to Recover Mercury from Hg^0-Rich Flue Gas. *Environ Sci Technol.* **54**, 11594-11601 (2020).
7. Xu H, *et al.* Enhancing the catalytic oxidation of elemental mercury and suppressing sulfur-toxic adsorption sites from SO_2-containing gas in Mn-SnS_2. *J Hazard Mater.* **392**, 122230 (2020).
